# Gait alteration strategies for knee osteoarthritis: a comparison of joint loading via generic and patient-specific musculoskeletal model scaling techniques

**DOI:** 10.1080/23335432.2019.1629839

**Published:** 2019-07-21

**Authors:** C M Dzialo, M Mannisi, K S Halonen, M de Zee, J Woodburn, M S Andersen

**Affiliations:** a Anybody Technology A/S, Aalborg, Denmark; b Department of Materials and Production, Aalborg University, Aalborg, Denmark; c School of Health and Life Sciences, Glasgow Caledonian University, Scotland, UK; d Department of Health Science and Technology, Aalborg University, Aalborg, Denmark

**Keywords:** Gait modifications, lateral insoles, medial compartment knee osteoarthritis, magnetic resonance imaging, musculoskeletal model, knee contact force, scaling, morphing

## Abstract

Gait modifications and laterally wedged insoles are non-invasive approaches used to treat medial compartment knee osteoarthritis. However, the outcome of these alterations is still a controversial topic. This study investigates how gait alteration techniques may have a unique effect on individual patients; and furthermore, the way we scale our musculoskeletal models to estimate the medial joint contact force may influence knee loading conditions. Five patients with clinical evidence of medial knee osteoarthritis were asked to walk at a normal walking speed over force plates and simultaneously 3D motion was captured during seven conditions (0°-, 5°-, 10°-insoles, shod, toe-in, toe-out, and wide stance). We developed patient-specific musculoskeletal models, using segmentations from magnetic resonance imaging to morph a generic model to patient-specific bone geometries and applied this morphing to estimate muscle insertion sites. Additionally, models were created of these patients using a simple linear scaling method. When examining the patients’ medial compartment contact force (peak and impulse) during stance phase, a ‘one-size-fits-all’ gait alteration aimed to reduce medial knee loading did not exist. Moreover, the different scaling methods lead to differences in medial contact forces; highlighting the importance of further investigation of musculoskeletal modeling methods prior to use in the clinical setting.

## 1. Introduction

Knee osteoarthritis (KOA) is a leading cause of global disability due to the irreversible deterioration of knee joint cartilage (Cross et al. 2014). Partial or total knee replacements (TKR) have proven to be effective treatments for end-stage KOA (Carr et al. 2012). However, as the age at which patients receive the replacement is decreasing (Losina et al. 2012; Goudie et al. 2017) and human life expectancy increasing, it is only natural that the rate of revision surgeries is also increasing (Pabinger et al. 2013; Chawla et al. 2017). It is known that estimated lifetime risk of revision (LTRR) is significantly higher for those patients under age 70, especially with respect to 50–54-year-old men with a 35% LTRR (Bayliss et al. 2017). It has been shown that patients undergoing TKR at an early age will have more wear of the implant than those of older patients (Fernandez-Fernandez and Rodriguez-Merchan 2015). Therefore, the need for non-surgical interventions to treat early-stage KOA is great; to delay the onset of late-stage KOA and ultimately the age at which joint replacement surgery may become a viable option.

Non-surgical interventions aimed at treating early-stage medial KOA such as lateral wedge insoles (LWI) and gait modifications (toe-in, toe-out, wide stance, trunk sway) have been introduced. However, the success of these treatments has not always been exclusive (Bennell et al. 2011; Hinman et al. 2012; Penny et al. 2013; Arnold 2016). Both toe-in and toe-out walking have shown to reduce knee adduction moment (KAM) on average (Shull et al. 2013a, 2013b; Hunt and Takacs 2014) but not all patients respond positively. Directing researchers to tailor treatments to the individual patient (Gerbrands et al. 2014; Shull et al. 2015; Favre et al. 2016). One such study has shown that more patient-specifically assigned toe-in and toe-out walking can better reduce the peak KAM, a controversial surrogate for the medial contact force (Creaby 2015; Manal et al. 2015; Richards et al. 2018), when compared to uniformly assigned modifications (Uhlrich et al. 2018).

The importance of patient-specific musculoskeletal modeling, especially for use in the clinical setting, has been highlighted (Fregly et al. 2012; Gerus et al. 2013; Clément et al. 2015). However, many gait alteration studies either (1) utilize biofeedback techniques which most often evaluate the gait in terms of KAM, and/or knee flexion moment (KFM) and furthermore rarely use patient-specific musculoskeletal models beyond simple linear scaling (Fregly 2007; Shull et al. 2013a; Ogaya et al. 2014; Miller et al. 2015; van Den Noort et al. 2015; Liu et al. 2016; Richards et al. 2017) or (2) have performed analysis on healthy subjects (Shull et al. 2011; Wheeler et al. 2011; Caldwell et al. 2013; van Den Noort et al. 2013; Miller et al. 2015; Halonen et al. 2017; Pizzolato et al. 2017; Uhlrich et al. 2018). This should raise the question on whether differences exist between linearly scaled models and patient-specific models with regards to the evaluation of knee contact forces. Would these differences between models lead to different conclusion on patient-specific interventions?

The objectives of this study were (1) to determine how gait alterations (LWI or gait modifications) influence knee loading through use of patient-specific musculoskeletal modeling, (2) identify which alteration minimizes medial contact force (MCF) at the individual and patient-group level, and (3) investigate if we reach the same conclusions using a simple linearly scaled (LS) model when compared to a non-linear magnetic resonance imaging (MRI)-based model.

## 2. Methods

### 2.1. Experimental data

#### 2.1.1. Patients

We recruited and studied five patients with a confirmed diagnosis of medial knee osteoarthritis according to American College of Rheumatology (Altman et al. 1986) and 50 years or older (Table 1). The study was approved by NHS Greater Glasgow and Clyde ethical committee (www.hra.nhs.uk 15-WS-0287 183203). Informed consent was obtained from all patients before data collection commenced. The study was conducted in accordance with the Declaration of Helsinki.

**Table 1.** Patient demographics.

Patient number	Age [yrs]	Mass [kg]	Height [cm]	BMI [kg/m^2^]	Tested leg	Varus angle [°]	Sex	Kellgren and Lawrence grade		
								medial	lateral	patella
1	64	74	156	30.41	R	##	F	4	2	3
2	60	112	184	33.08	L	4	M	4	3	3
3	56	90	163	33.87	R	5	F	4	2	4
4	74	89	165.5	32.49	R	7	M	4	2	1
5	58	71.2	167.5	25.38	L	8	M	4	2	3

## Data missing.

#### 2.1.2. MRI imaging

**Figure 1.**
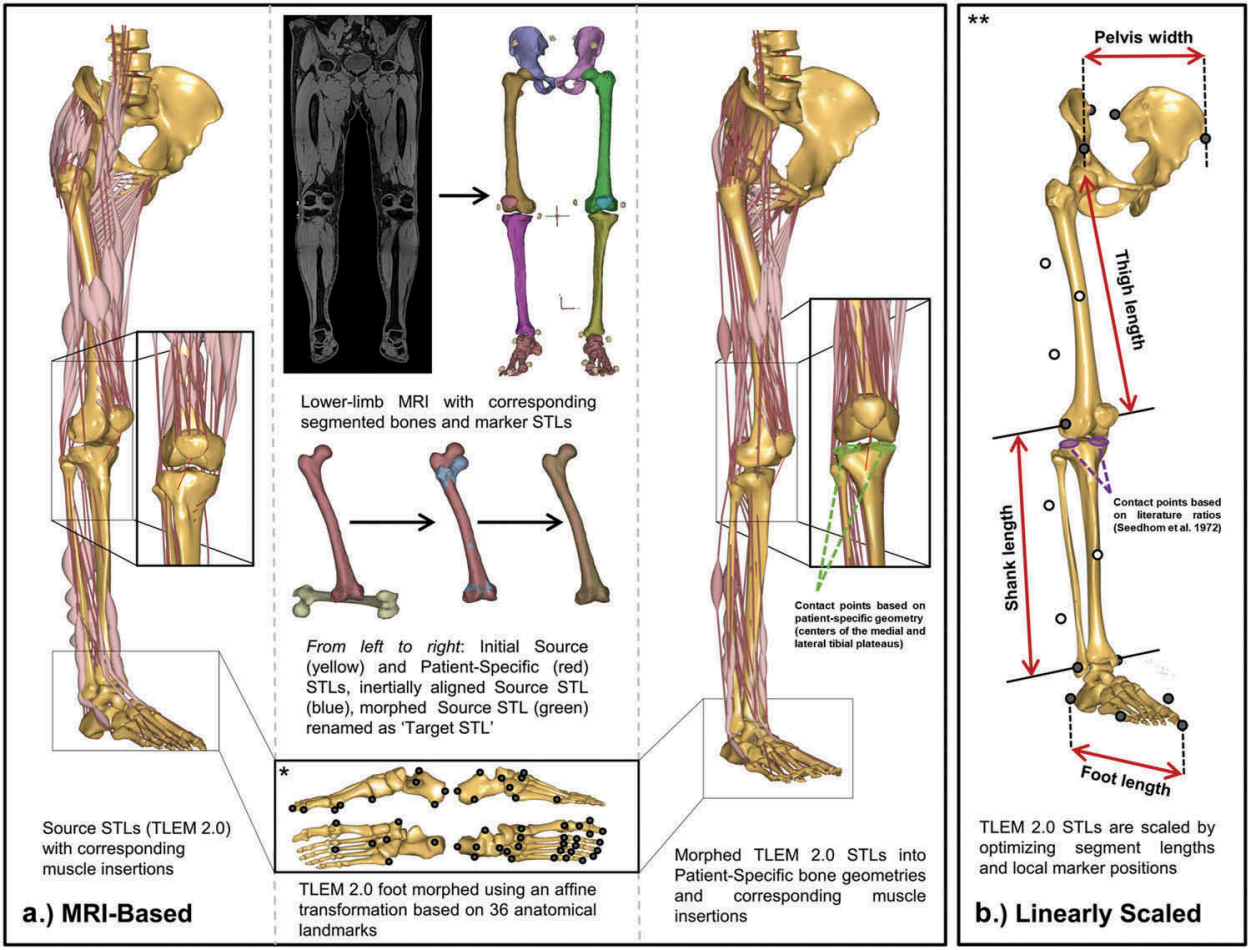
Structure for patient-specific models using (A) MRI-based morphing and (B) linear scaling techniques. *Graphic is adapted from supplementary figure B in Halonen et al. (2017). **Graphic is adapted from Figure 2 in Lund et al. (2015) substituting bone geometries for TLEM 2.0 STLs.

Each patient underwent a lower-limb MRI using a 3T Siemens Prisma scanner and Peripheral Angio 36 coil. Prior to the scan, 20 PinPoint® markers (Beekley Medical®, Bristol Connecticut) were placed in key anatomical landmarks corresponding to the motion capture marker setup (Figure 1(a)). The lower limb scan (total acquisition time 15 min) was obtained in three segments. For each segment, the table was moved further into the MRI bore, and a T1 weighted Vibe-Dixon sequence was acquired in the transverse plane (resolution 320 square pixels, slice thickness 1.4 mm, gap thickness 0 mm, and field of view [FOV] 440 mm × 440 mm). The overlapping images were stitched together (Figure 1(a)) to achieve a composed image and then reconstructed in the sagittal and coronal planes.

#### 2.1.3. Lateral wedge insoles

**Figure 2.**
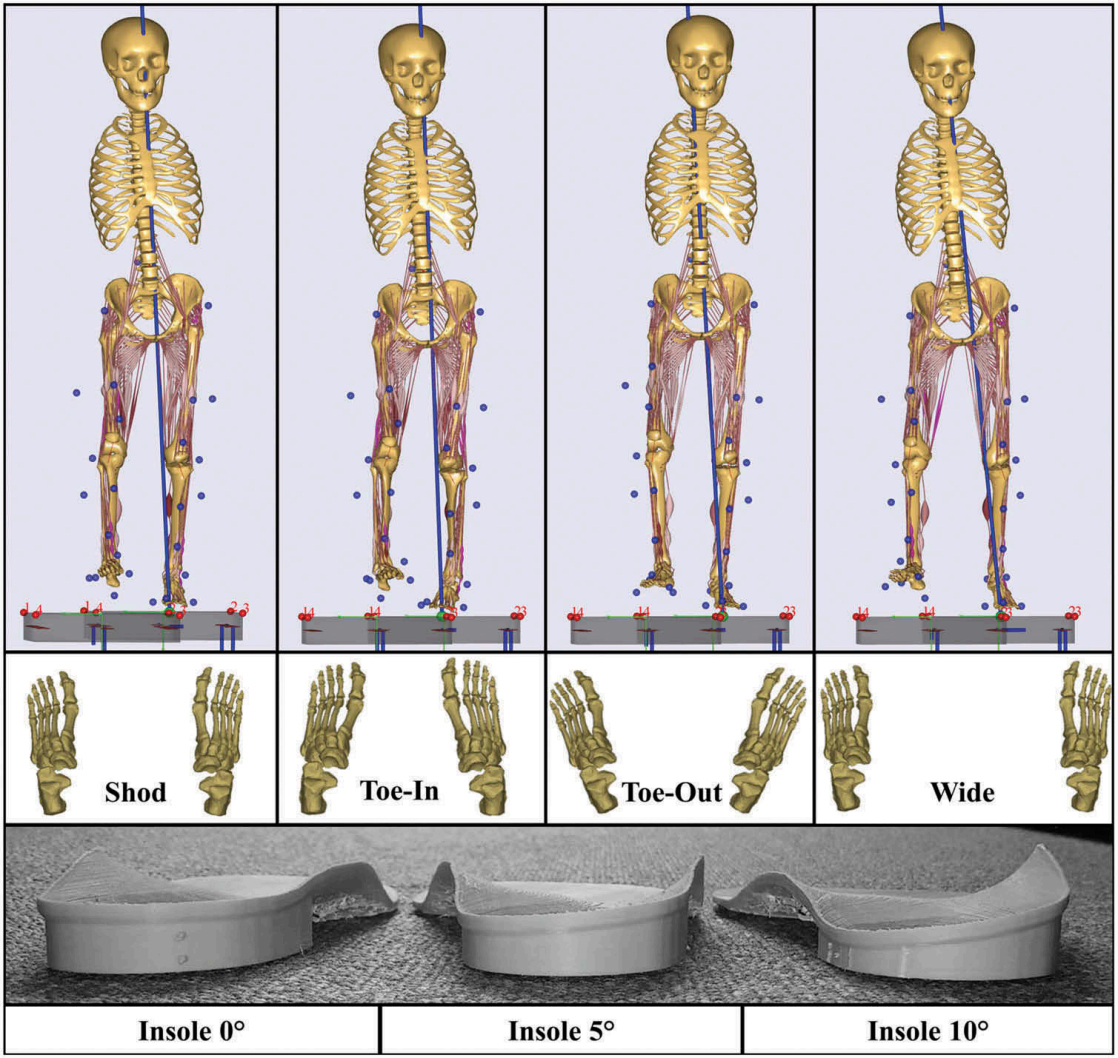
Gait modification techniques (baseline-shod, toe-in, toe-out, and wide stance walking) and lateral-wedged insoles (0°, 5°, and 10°).

Podotech Foot Impression Boxes (A. Algeo Ltd, Liverpool, United Kingdom) were used to obtain surface geometry of the patients’ feet. Each impression was scanned using a Sense 3D Scanner (3D Systems, Rock Hill, SC, US) and imported into Rhinoceros 3D V5 software (Robert McNeel & Associate, Barcelona, Spain) where three sets of LWIs were designed (inclines: 0°, 5°, and 10°). Finally, the insoles (Figure 2) were manufactured using an Airwolf 3D HDX 3D printing system (Airwolf 3D printers, Costa Mesa, USA).

#### 2.1.4. Gait alterations and motion capture

Kinematic data were collected in the Human Performance Lab of Glasgow Caledonian University (GCU, Glasgow, UK) using a 14 Qualisys Opus camera system (Qualisys AB, Gothenburg, Sweden) sampling at 120 Hz. Simultaneously, ground reaction forces were recorded using force plates (9286BA, Kistler Group, Winterthur, Switzerland) sampling at 2000 Hz. Thirty-two motion capture markers were placed bilaterally on key anatomical landmarks. A standing reference trial of each patient was captured prior to dynamic trials. The patients were asked to perform seven gait variations (Figure 2): normal shod walking (‘*Shod*’), three walking trials with different degrees of LWI (‘*Insoles-0ʹ*, ‘*Insoles-5ʹ*, ‘*Insoles-10ʹ*), and three gait modifications with toes turned slightly inwards (‘*toe-in’*), toes turned slightly outwards (‘*toe-out’*) and walking with a wider stance (‘*wide*’) at a self-selected walking speed (Supplementary Table 1). Additionally, we ensured that the second force plate was contacted with the symptomatic leg for each trial by adjusting the patients’ starting position. For all gait trials, patients wore neutral posted training shoes provided by the lab that were adapted in a previous study to show foot markers (Telfer et al. 2013). For each gait modification, the patients were taught the necessary movements verbally and given sufficient practice time until they felt comfortable. The patients were asked to perform five trials of each gait variation, however, if significant pain arose, this was decreased to three trials.

### 2.2. Musculoskeletal models

#### 2.2.1. Lower-limb model

Patient-specific musculoskeletal models were created using Anybody Modeling System (AMS) version 7.1 (Anybody Technology A/S, Aalborg, Denmark) (Damsgaard et al. 2006) and the generic human body model from the AnyBody Managed Model Repository (AMMR) version 1.6. The arms were excluded and the legs were updated to utilize the Twente Lower Extremity Model version 2.0 (TLEM 2.0) dataset (Carbone et al. 2015). The model consisted of 13 segments (head, trunk, pelvis, and right/left thigh, patella, shank, talus, and foot) connected by hinge joints (neck, tibiofemoral, patellofemoral, talocrural, and subtalar) and spherical joints (hip and lumbar spine vertebrae). Muscle-tendon units were modeled using Hill-type one-dimensional actuators running from origin to insertion through via-points and wrapping surfaces which were defined either through (1) linear scaling methods or (2) analytical surface fits of the patient-specific bone geometries (MRI morphed) respectively.

#### 2.2.2. MRI-based model

MRI-based models (Figure 1(a)) were created of each subject starting with the manual segmentation of the water composed MRI scans using Mimics 19.0 Research (Materialise, Leuven, Belgium). The MRI markers segmentations were exported as individual stereolithography (STL) files and later paired with the experimental motion capture skin markers during the kinematic trials of the MRI-based models. Then, the pelvis, femur, tibia, patella, talus, and foot bones were segmented and saved as patient-specific STLs. These patient-specific STLs were used to morph cadaver-based STLs from the TLEM v 2.0 (Carbone et al. 2015) utilizing inertial alignment and morphing tools in Mimics (Marra et al. 2015; Halonen et al. 2017). These newly morphed STLs have matching point numbers as those in the AMMR. Geometric morphing in AMS was carried out in a way similar to that performed in Halonen et al. (2017), through means of (1) an affine transformation, (2) tri-harmonic radial basis function interpolation, and (3) a reverse rigid-body transformation. Each foot was morphed using 36 anatomical landmarks and an affine transformation to capture the patient-specific shape and size of the foot. The tibiofemoral, patellofemoral, ankle, and subtalar joints were modeled as revolute joints, and the hip joint was modeled as a sphere. All were defined with analytical shape fitting methods (cylinder and sphere, respectively) utilizing patient-specific geometries selected in 3-Matic 11.0 Research (Materialise, Leuven, Belgium). Pelvis width, thigh, shank, and foot lengths were calculated from joint to joint distances from the geometrical morphing and used as input variables for muscle strength scaling.

#### 2.2.3. Linearly scaled model

Five patient models were also established using the most common linear scaling method (Figure 1(b)) in AMS based off skin markers trajectories (Lund et al. 2015). The skin marker trajectories were used to optimize selected marker positions and the scaling of the pelvis width, thigh, shank, and foot length. These segment lengths were later used as input variables for muscle strength scaling. The nonlinear least-square optimization method used was introduced by Andersen et al. (2010) and minimized the least-squares difference between experimental motion capture skin markers and the model markers placed at corresponding locations in AMS during the standing reference trial. Marker positions on bony landmarks (black markers in Figure 1(b)) remained fixed while markers on the thigh and shank segments (white markers in Figure 1(b)) were optimized.

#### 2.2.4. Muscle modeling

Both model types utilized Hill type muscles models from the TLEM 2.0 dataset using the length-mass-fat scaling law to estimate the muscle’s isometric strength based on body fat percentage (BMI), segment mass, and segment length (Rasmussen et al. 2005). The size of each segment is determined based on the length and the mass in two directions, and the length variable along the third. In addition, the law takes BMI into account before estimating the isometric strength of the muscles. We solved the muscle recruitment problem by minimizing a 3^rd^ order polynomial cost function $\left(G\right)$, with respect to dynamic equilibrium equations, and allowing the muscles $\left(M\right)$ to each generate an individual $i^{th}$ force $f_{i}^{\left(M\right)}$ no greater than the $i^{th}$ instantaneous strength $N^{i}$ only by pulling, not pushing (Equation 1). Similar to past researchers (Marra et al. 2015; Halonen et al. 2017), a muscle volume normalization factor (Happee and Van Der Helm 1995) was introduced $v_{i}$; accounting for the force subdivision among split and non-split muscles.

$$\underset{f}{\min}G\left(f\right)=\;\overset{n^{\left(M\right)}}{\underset{i=1}{\sum }}v_{i}{\left(\frac{f_{i}^{\left(M\right)}}{N_{i}^{\left(M\right)}}\right)}^{3}$$

**s.t**.

(1) Cf=d

$$0\;\leq \;f_{i}^{\left(M\right)}\;\leq \;N_{i}^{\left(M\right)},\;i=1,\ldots ,\;n^{\left(M\right)}.$$

where $n^{\left(M\right)}$ is the number of muscles. The equilibrium equations consist of a coefficient matrix (C) of all unknown muscle and joint reaction forces (f) and a right-hand side containing all inertia forces and applied loads (d).

### 2.3. Data processing and statistical analysis

For both model types, three trials with the most consistent walking speed for each gait alteration were run through an over-determinate kinematic solver (Andersen et al. 2009) to compute joint angle trajectories. Subsequently, joint angles and ground reaction forces and moments were used as inputs to drive the inverse dynamics model, resulting in estimates of muscle forces, joint reaction forces, and joint moments. Knee joint reaction forces and moments were extracted from the inverse dynamics model in a tibial coordinate system (Grood and Suntay 1983). For the MRI-based model, bony landmarks from the patient-specific bone geometries were manually marked and exported as separate STLs using 3-Matic software, then post processed using custom written MATLAB code to determine mean location for use in AMS. For the right leg, the proximal-distal (PD)-axis points from the talocrural joint center (Parra et al. 2012) to the midpoint between the medial and lateral tibia edges, the medial-lateral (ML)-axis is orthogonal to the PD-axis and points towards the lateral tibia edge (medial for left knee), and the anterior-posterior (AP)-axis is the cross product of the PD and ML axis, pointing anteriorly. The LS-based model used the scaled bony landmarks from the TLEM 2.0 model to define the tibial coordinate system using the same definition outlined above. The medial and lateral contact forces were calculated by setting up a force equilibrium in the frontal plane using the total knee contact force, abduction/adduction moment, and moments arms measured on the patient-specific geometry from the tibial origin to the centers of the medial and lateral tibia plateaus. For the linearly scaled models, these moments arms were calculated based on relationships reported by Seedhom et al. (1972). The primary parameters were MCF and KAM; while knee flexion angle (KFA), knee flexion moment (KFM), lateral (LCF), and total contact forces (TCF) were also examined (Supplementary Tables 2–4). For both models, the trial data were resampled from heel strike to toe off and mean (standard deviations) were calculated (n = 3) for each gait alteration for all five patients. Patient means (standard deviations) were also calculated by normalizing the data with respect to percent body weight (%BW) for forces and percent body weight height (%BW·BH) for moments.

Descriptive statistics were calculated for peak MCF, MCF impulse, peak KAM, and KAM impulse for both the patients as a group (Table 2) and the patients individually (Table 3). This was done for both models, LS and MRI-based, allowing us to examine the differences between modeling techniques. We performed 4 one-way repeated measures ANOVAs (two MCF parameters obtained using two modeling techniques) to determine if any significant differences existed between baseline shod walking and the various gait alterations. This was done two ways: (1) looking at how each gait alteration influenced the patient-group compared to baseline shod and (2) comparing the gait alteration that results in the greatest load reduction for an individual patient and how this compares to their baseline shod walking. Due to the small sample size and multiple comparisons, post-hoc tests were corrected using Bonferroni adjustments (α = 0.05). Prior to performing statistical analysis, the paired differences were tested for normality using Shapiro-Wilk tests. Statistics were calculated in SPSS version 25 (IBM, New York, USA) with α = 0.05.

**Table 2.** Comparison of MRI-based and Linearly scaled models: Patient-group mean ± standard deviation peak medial MCF, MCF impulses, peak KAM, and KAM impulse (n = 5 patients, m = 3 trials for each gait) data of each gait alteration.

Outcome parameter	Shod	Insole_0	Insole_5	Insole_10	ToeIn	ToeOut	Width
**Linear scaling**							
Peak KAM [%BW*BH]	3.44 ± 0.59	3.39 ± 0.62	3.39 ± 0.56	3.39 ± 0.62	**3.08 ± 0.58**	3.27 ± 0.70	3.12 ± 0.72
KAM Impulse [%BW*BH*s]	1.50 ± 0.31	1.49 ± 0.34	1.50 ± 0.32	1.53 ± 0.38	1.38 ± 0.38	1.52 ± 0.37	**1.38 ± 0.34**
Peak Medial Comp Force [%BW]	240.50 ± 29.01	239.06 ± 32.60	237.40 ± 29.44	243.80 ± 30.68	227.73 ± 27.00	232.61 ± 25.94	**226.19 ± 35.22**
Medial Comp Impulse [%BW*s]	115.07 ± 19.74	114.23 ± 19.12	114.08 ± 16.73	117.94 ± 22.64	114.63 ± 19.70	118.24 ± 22.61	**112.50 ± 20.77**
**MRI scaling**							
Peak KAM [%BW*BH]	2.92 ± 0.30	2.87 ± 0.26	2.94 ± 0.42	2.89 ± 0.39	**2.70 ± 0.37**	2.93 ± 0.30	2.81 ± 0.30
KAM Impulse [%BW*BH*s]	1.37 ± 0.43	1.37 ± 0.38	1.39 ± 0.42	1.39 ± 0.39	**1.26 ± 0.41**	1.41 ± 0.36	1.28 ± 0.32
Peak Medial Comp Force [%BW]	234.21 ± 40.29	233.11 ± 44.86	237.25 ± 39.70	237.34 ± 41.04	**221.67 ± 33.69**	238.66 ± 42.68	233.14 ± 48.88
Medial Comp Impulse [%BW*s]	114.59 ± 17.94	**114.19 ± 14.57**	116.71 ± 13.83	118.04 ± 17.57	118.21 ± 26.78	121.06 ± 24.68	116.46 ± 23.08

**Bold** indicates value with the greatest reduction.

**Table 3.** Comparison of MRI-based and Linearly scaled (LS) models: Individual patient peak KAMs, KAM impulses, peak MCF, and MCF impulses (mean ± standard deviation from n = 3 trials) of each gait alteration.

## 3. Results

**Figure 3.**
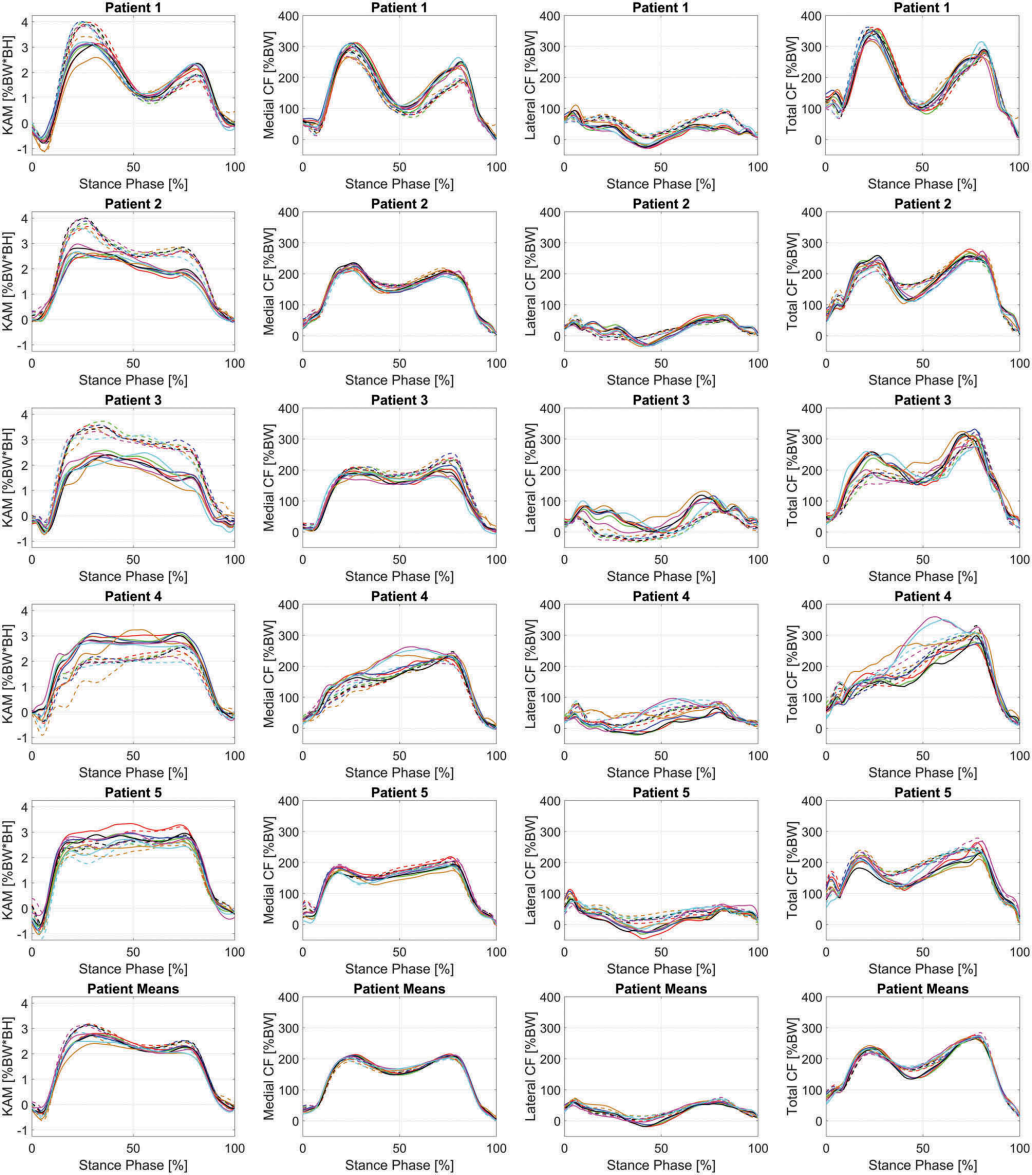
Comparison of MRI-based (solid lines) and Linearly scaled (dashed-lines) models in terms of mean knee adduction moment (KAM), medial compressive forces (MCF), and lateral compressive forces (LCF) for each individual patient (n = 3 trials) during stance phase of various gait alterations: shod (black), 0° insole (green), 5° insole (red), 10° insole (blue), Toe-in (orange), Toe-out (magenta), and width (cyan). In addition, subject means are displayed in the bottom row (n = 5 subjects, 3 trials each).

Differences exist between the LS and MRI-based models (Figure 3) for the outcome parameters. The gait alteration that produced the greatest reduction in each outcome parameter (peak and impulse, separately) differed with respect to the individual patients and the patient-group (Table 4). At most, only two out of five patient-specific alteration effects match what was achieved through patient-group analysis. Interestingly, the LS models achieved greater knee flexion angles and moments throughout the stance phase compared to the MRI-models (Supplementary Figure 1). Average differences, calculated between LS-based and MRI-based models, for KFA ROM (8.75 ± 2.57°), peak KFM (0.81 ± 0.47 %BW*BH), and KFM impulse (0.044 ± 0.084 %BW*BH*s) were extracted (Supplementary Table 2). Moreover, both on a group and individual patient level, for both modeling techniques, the gait alteration that achieved the greatest reduction in KFM (peak and impulse) measures was comparable to our KAM findings (Supplementary Tables 2 and 4).

**Table 4.** Comparison of MRI-based and Linearly scaled models for gait alteration that results in the greatest average reduction in load (MCF peak and impulse, separately) looking at the individual patients and as a group of patients.

	*Gait Alteration with greatest reduction in:*			
	Peak MCF		MCF Impulse	
Patient	LS	MRI	LS	MRI
*1*	Toe-In	Toe-In	Toe-Out	Toe-In
*2*	Width	Width	Width	Width
*3*	Width	Toe-Out	**none**	**none**
*4*	Toe-Out	Insoles 5°	Insoles 5°	Insoles 0°
*5*	Toe-In	Toe-In	Width	Toe-In
***Patient-group***	**Width**	**Toe-In**	**Width**	**Insoles 0**

Furthermore, the gait alteration effects differed between model scaling and morphing techniques. In fact, less than 50% of the time (4 out of the 10 situations) do the LS and MRI-based models result in the same reduction in knee loading when examining patients individually (Table 4). In addition, we found by assigning patient-specific gait alterations and comparing the resulting parameters to their normal shod walking trial, resulted in significant decreases in MCF peaks during the stance phase for both model types, and MCF impulse for the MRI-based model (Table 5). However, this was not always the case when assigning a uniform gait alteration to the patient-group.

**Table 5.** Pairwise comparison results from post-hoc testing of two-way repeated-measures ANOVA investigating the mean difference of **peak medial contact force** between baseline (shod) and selected treatment outcome based for the Linearly scaled and MRI-based models (%BW).

					95% Confidence Interval for Difference	
Baseline (I)	Gait alteration selection applied to group (J)	Mean Difference (I-J) [%BW]	Std. Error	P-value	Lower Bound	Upper Bound
**LS-Model**						
Shod	0° LW	1.440	3.442	1.000	−11.792	14.673
	5° LW	3.099	5.496	1.000	−18.034	24.232
	10° LW	−3.303	5.622	1.000	−24.917	18.312
	Toe-in	12.765	4.735	0.487	−5.441	30.971
	Toe-out	7.893	6.896	1.000	−18.622	34.408
	Wide	14.313	5.730	0.716	−7.717	36.343
	**Based on Individual Patient**	20.514*	**4.486**	**0.012**	**3.267**	**37.761**
**MS-Model**						
Shod	0° LW	1.106	3.789	1.000	−13.461	15.672
	5° LW	−3.038	6.048	1.000	−26.293	20.217
	10° LW	−3.126	4.911	1.000	−22.006	15.754
	Toe-in	12.538	6.201	1.000	−11.305	36.381
	Toe-out	−4.442	6.639	1.000	−29.967	21.082
	Wide	1.072	6.496	1.000	−23.905	26.049
	**Based on Individual Patient**	18.748*	**4.442**	**0.024**	**1.669**	**35.827**

The bold rows, ‘Based on Individual Patient’, examine the gait alterations select individually for each patient, based on the greatest reduction in load, rather than applying the same treatment to the entire patient group. * denotes that result is significant to (α = 0.05)

**Table 6.** Pairwise comparison results from post-hoc testing of two-way repeated-measures ANOVA investigating the mean difference of **medial contact force impulse** between baseline (shod) and selected treatment outcome based for the Linearly scaled and MRI-based models (%BW*s).

					95% Confidence Interval for Difference	
Baseline (I)	Gait alteration selection applied to group (J)	Mean Difference (I-J) [%BW*s]	Std. Error	P-value	Lower Bound	Upper Bound
**LS-Model**						
Shod	0° LW	0.832	2.061	1.000	−6.791	8.456
	5° LW	0.984	2.669	1.000	−8.889	10.857
	10° LW	−2.877	2.989	1.000	−13.933	8.179
	Toe-in	0.432	2.459	1.000	−8.664	9.529
	Toe-out	−3.171	2.323	1.000	−11.764	5.421
	Wide	2.562	1.925	1.000	−4.558	9.683
	**Based on Individual Patient**	**4.435**	**2.142**	**0.057**	**−0.159**	**9.029**
**MS-Model**						
Shod	0° LW	0.396	1.645	1.000	−5.689	6.481
	5° LW	−2.121	2.236	1.000	−10.391	6.149
	10° LW	−3.454	2.409	1.000	−12.364	5.456
	Toe-in	−3.624	2.800	1.000	−13.982	6.733
	Toe-out	−6.466	2.496	0.449	−15.700	2.767
	Wide	−1.873	2.552	1.000	−11.316	7.569
	**Based on Individual Patient**	3.241*	**1.497**	**0.048**	**0.031**	**6.452**

The bold rows, ‘Based on Individual Patient’, examine the gait alterations select individually for each patient, based on the greatest reduction in load, rather than applying the same treatment to the entire patient group. * denotes that result is significant to (α = 0.05)

### 3.1. MRI-based model

The MRI-based model predicted on average (Table 2 and Supplementary Table 2) that ‘*toe-in*’ walking would achieve the greatest reduction in peak KAM, KAM impulse, and peak MCF; while walking with ‘*insole-0ʹ* resulted in the lowest peak TCF, TCF impulse, and MCF impulse values. On a patient-specific basis (Table 3 and Supplementary Table 3), Patient 1 had the greatest reductions in MCF and TCF impulse values occurred during ‘*toe-out*’ walking. Similarly, ‘*toe-in*’ walking again obtained the lowest peak MCF. For Patient 2, MCF and TCF peak and impulse values reduced most during ‘*wide*’ walking. Patient 3 achieved the lowest peak MCF with a ‘*toe-out*’ gait alteration. However, none of the gait alteration techniques reduced the MCF impulse for Patient 3. The greatest decrease in peak MCF and TCF for Patient 4 occurred with ‘*insoles-5ʹ*. The MCF and TFC impulses were both reduced walking with ‘*insoles-0ʹ*. For Patient 5, although a decrease in MCF impulse was observed during ‘*toe-in’* walking; all alterations increased the TCF impulse. Finally, the peak MCF and TCF had the greatest decrease during ‘*toe-in*’ walking. Moreover, when applying a uniform gait alteration to the patient-group, no significant reduction in peak MCF and MCF impulse exist when compared to baseline-shod walking. The only significant reduction occurred when applying patient-specifically assigned gait alterations (18.748 ± 4.442 %BW and 3.241 ± 1.497 %BW*s) for peak and impulse, respectively.

### 3.2. Linearly scaled model

The LS model predicted on average (Table 2 and Supplementary Table 2) that ‘*wide’* walking would achieve the lowest KAM impulse, peak MCF, peak TCF, and MCF impulse values, and second lowest peak KAM. On a patient-specific basis (Table 3 and Supplementary Table 3), Patient 1 had the greatest decrease in impulse values occurred during ‘*toe-out*’ walking. While, ‘*toe-in*’ walking obtained the lowest peak MCF. For Patient 2, ‘*wide*’ walking reduced the MCF and TCF peak and impulse values the most. Patient 3 achieved the lowest peak MCF when walking with a ‘*wide*’ gait alteration. However, similar to the MRI-based model, a gait alteration that reduced MCF impulse for Patient 3 did not exist. The greatest decrease in peak MCF for Patient 4 occurred when walking with ‘*toe-out*’, while the MCF and TFC impulses were both reduced walking with ‘*insoles-5ʹ*. For Patient 5, although a decrease in MCF impulse was observed during ‘*wide*’ walking; all alterations increased the TCF impulse. In addition, the peak MCF and TCF had the greatest reductions during ‘*toe-in*’ walking. Finally, similar to the MRI-based model, when applying a ‘one-size-fits-all’ gait alteration, there were no significant differences when compared again baseline walking for peak MCF and MCF impulse (Tables 5 and 6). The only significant reduction occurred in peak MCF when applying patient-specifically assigned gait alterations (20.514 ± 4.486 %BW). Although the uniformly applied *‘wide’* gait alteration for the patient-group had the greatest reductions in peak and MCF impulse, statistically they were not significant. Additionally, while a significant difference did not exist for MCF impulse, the patient-specific-assigned gait alterations did achieve the greatest reduction with a p-value of 0.057. This low p-value tells us that this data is unlikely to accept the null hypothesis.

## 4. Discussion

The purpose of this study was to first investigate how knee loading is influenced by various gait alterations using patient-specific musculoskeletal modeling. The resulting peak and impulse MCF were then examined to determine which gait alteration produced the greatest reduction with respect to the patients individually and as a group. Furthermore, we developed patient-specific musculoskeletal models using two scaling methods: simple linear scaling and MRI-based morphing, to discover if varying the modeling approach leads to the identification of different MCF reducing gait alterations.

Our results confirmed the conflicting literature evidence behind whether these interventions actually work (Bennell et al. 2011; Penny et al. 2013; Arnold 2016; Uhlrich et al. 2018), by furthering the evidence that these inconsistencies may arise due to the fact that a uniform alteration is often assigned to a patient-group (Shull et al. 2013a; Hunt and Takacs 2014) rather than more individualized alterations (Uhlrich et al. 2018). Additionally, based on the results of this study, we recommend that care should be taken when selecting a modeling technique because this influences the contact moment arms, known to linearly impact medial-lateral contact forces (Lerner et al. 2015; Saliba et al. 2017), and thus overall identification of the best gait alteration. It is known estimated medial and lateral contact forces are sensitive to contact locations and tibiofemoral alignment (Saliba et al. 2017). And furthermore, that applying individualized TF alignment and contact locations will lead to better medial and lateral force predictions compared to generic parameters (Lerner et al. 2015). The fact that the LS model (1) preserves the knee alignment of the TLEM cadaver data during the scaling process and (2) has contact moment arms adopted from the literature (Seedhom et al. 1972), may lead to the possibility of it not representing true MCF and LCFs. This is of great importance considering so many studies stray beyond simple linear scaling (Fregly 2007; Shull et al. 2013a; Ogaya et al. 2014; Miller et al. 2015; van Den Noort et al. 2015; Liu et al. 2016; Richards et al. 2017).

Two of the main limitations of this study were the small sample size and not randomizing the order of gait alterations, although beyond the scope of this study, this restricted detection of meaningful clinical results. Additionally, the patellofemoral and tibiofemoral joints were simplified as hinges. However, it has been shown that the tibiofemoral contact force predictions do not differ significantly from those of a more complex knee model utilizing force-dependent kinematics (Marra et al. 2015). It should be noted that Marra et al. modeled a subject with a TKR, making this a difficult generalization (comparison of hinge and FDK results), especially for patients with KOA whom often have abnormal knee kinematics. Also, the contact moment arms were assumed constant with respect to knee flexion for both modeling techniques which may be a limitation for patients with KOA, especially those with large osteophytes potentially altering the TF contact location. We speculate that the differences between the MRI-based and LS models in terms of KFA, KAM, KFM, and MCF could be attributed to how the knee joint is modeled and preserved during different modeling techniques. More specifically, how the knee is aligned and the location of the medial and lateral compartment contacts (Lerner et al. 2015; Lund et al. 2015; Moissenet et al. 2017). Furthermore, only four patients were able to perform maximum isometric strength measurements; so, for sake of model consistency, we used generic muscle-tendon parameters. We recommend that for future work a sensitivity study is conducted between the two models by examining the contact moment arm, muscle strength, knee alignment, and bony geometry influencing muscle insertion points. Lastly, this study only investigated the immediate effect of gait alteration techniques on knee contact force, a long-term study with gradual introduction of LWI and a training program for gait modification techniques may change the results (Lewinson et al. 2016). In addition, the gait modifications were taught during a single training session. Multiple training sessions, practicing at home, and biofeedback (Shull et al. 2013b; Hunt and Takacs 2014; Richards et al. 2017) might prove more beneficial to fully retain a new pattern of walking (Hunt and Takacs 2014).

In conclusion, this study explored how patients with medial KOA are influenced by gait alterations aimed to reduce peak and impulse MCF using patient-specific musculoskeletal modeling. We did not find a ‘one-size-fits-all’ gait alteration effect, suggesting the importance of individually assigned interventions. However, this study does not aim to make clinical recommendations; but rather to establish groundwork for future studies to identify gait alterations that can achieve a desired MCF. Most importantly, we discovered that different patient-specific musculoskeletal model scaling techniques can produce different MCF results. Future studies should examine which model scaling technique better predicts reality through additional model validation. Additionally, treatment selections should be tested longitudinally to understand if the model designated gait alterations actually result in better clinical outcomes, i.e., in terms of cartilage health and patient satisfaction. Future work should also include finite element analysis to estimate tissue loads and relate these to measurements of cartilage health following long-term use of the identified best patient treatments. Hereby, researchers can better understand whether parameters that closely represent the tissue response, can better identify the true patient response to the selected treatment and how that may relate to long-term outcomes.

## Supplementary Material

Supplemental Material
