# Supplementary material for: Gait alteration strategies for knee osteoarthritis: a comparison of joint loading via generic and patient-specific musculoskeletal model scaling techniques
Source: Int Biomech. 2019 Jul 21;6(1):54–65. doi: 10.1080/23335432.2019.1629839 (PMC7857308; doi:10.1080/23335432.2019.1629839)
Supplement: Supplemental Material [file TBBE_A_1629839_SM1759.zip › Supplementary_Tables_final.docx]

**Supplementary Table 1.** Patient mean ± standard deviation (n=3) walking speed (m/s) for each gait alteration technique. Calculated from mean y-component velocity (along the force plate runway) of the pelvis markers (LPSIS, RPSIS, LASIS, and RASIS), and averaged from heel strike on force plate 1 to toe-off of force plate 2.

|  | Shod | Insole 0° | Insole 5° | Insole 10° | Toe In | Toe Out | Width |
| --- | --- | --- | --- | --- | --- | --- | --- |
| Patient 1 | 1.34 ± 0.04 | 1.37 ± 0.02 | 1.39 ± 0.02 | 1.38 ± 0.04 | 1.36 ± 0.02 | 1.37 ± 0.03 | 1.41 ± 0.04 |
| Patient 2 | 1.27 ± 0.03 | 1.26 ± 0.04 | 1.28 ± 0.02 | 1.29 ± 0.04 | 1.23 ± 0.02 | 1.24 ± 0.04 | 1.27 ± 0.01 |
| Patient 3 | 0.96 ± 0.04 | 0.95 ± 0.03 | 0.98 ± 0.04 | 0.93 ± 0.01 | 0.86 ± 0.05 | 0.86 ± 0.03 | 0.88 ± 0.03 |
| Patient 4 | 0.82 ± 0.04 | 0.88 ± 0.05 | 0.86 ± 0.03 | 0.86 ± 0.02 | 0.81 ± 0.05 | 0.82 ± 0.04 | 0.81 ± 0.04 |
| Patient 5 | 1.14 ± 0.04 | 1.12 ± 0.05 | 1.16 ± 0.06 | 1.19 ± 0.06 | 1.14 ± 0.04 | 1.16 ± 0.02 | 1.18 ± 0.03 |

**Supplementary Table 2.** Comparison of MRI-based and Linearly scaled models: Mean ± standard deviations of knee flexion angle range of motion (ROM), peak knee flexion moment (KFM), KFM impulse, total and lateral compartmental contact peak forces and impulses (n=5 patients, m=3 trials for each gait) data of each gait alteration.

| **Linearly scaled** | | | | | | | |
| --- | --- | --- | --- | --- | --- | --- | --- |
| **Names** | **Shod** | **Insole 0°** | **Insole 5°** | **Insole 10°** | **Toe-In** | **Toe-Out** | **Width** |
| Knee Flexion Angle ROM [°] | 48.43 ± 0.75 | 47.10 ± 0.93 | 48.43 ± 1.58 | 46.35 ± 1.15 | 47.15 ± 1.48 | 48.93 ± 1.25 | **43.05 ± 2.32** |
| Peak KFM [%BW*BH] | 3.91 ± 0.19 | 4.00 ± 0.32 | 3.98 ± 0.08 | 4.05 ± 0.16 | **3.43 ± 0.18** | 3.83 ± 0.57 | 4.02 ± 0.29 |
| KFM Impulse [%BW*BH*s] | 0.98 ± 0.03 | 0.97 ± 0.02 | 0.96 ± 0.03 | 0.94 ± 0.08 | **0.89 ± 0.03** | 0.95 ± 0.10 | 0.93 ± 0.04 |
| Peak Lateral Contact Force [%BW] | **73.87 ± 13.32** | 76.00 ± 15.40 | 79.12 ± 19.64 | 79.38 ± 14.87 | 83.78 ± 14.27 | 78.97 ± 11.62 | 85.18 ± 13.47 |
| Peak Total Contact Force [%BW] | 298.69 ± 43.49 | 294.22 ± 47.67 | 294.03 ± 43.80 | 301.34 ± 45.75 | 292.07 ± 38.71 | 299.43 ± 28.90 | **291.52 ± 43.47** |
| Lateral Contact Impulse [%BW*s] | 24.80 ± 11.61 | 24.21 ± 11.44 | **23.75 ± 8.57** | 25.68 ± 10.25 | 31.36 ± 13.13 | 26.66 ± 13.41 | 29.28 ± 14.98 |
| Total Contact Impulse [%BW*s] | 139.87 ± 23.43 | 138.44 ± 20.03 | **137.83 ± 14.48** | 143.62 ± 21.90 | 146.00 ± 20.87 | 144.89 ± 26.74 | 141.78 ± 26.76 |
|  |  |  |  |  |  |  |  |
| **MRI-based** | | | | | | | |
| **Names** | **Shod** | **Insole 0°** | **Insole 5°** | **Insole 10°** | **Toe-In** | **Toe-Out** | **Width** |
| Knee Flexion Angle ROM [°] | 39.67 ± 1.15 | 38.29 ± 1.28 | 39.19 ± 1.03 | 37.10 ± 1.06 | 38.02 ± 1.35 | 40.62 ± 1.02 | **35.28 ± 1.66** |
| Peak KFM [%BW*BH] | 3.11 ± 0.21 | 3.09 ± 0.17 | 3.14 ± 0.13 | 3.15 ± 0.23 | **2.62 ± 0.23** | 3.18 ± 0.38 | 3.24 ± 0.17 |
| KFM Impulse [%BW*BH*s] | 0.92 ± 0.02 | 0.93 ± 0.02 | 0.91 ± 0.02 | 0.91 ± 0.07 | **0.80 ± 0.04** | 0.93 ± 0.06 | 0.91 ± 0.03 |
| Peak Lateral Contact Force [%BW] | 82.49 ± 24.78 | **78.63 ± 22.72** | 88.63 ± 27.68 | 87.93 ± 27.70 | 99.89 ± 29.08 | 86.74 ± 17.05 | 87.22 ± 19.56 |
| Peak Total Contact Force [%BW] | 290.98 ± 42.36 | **289.37 ± 45.38** | 298.72 ± 39.91 | 293.68 ± 48.55 | 291.36 ± 49.15 | 306.02 ± 42.82 | 297.38 ± 49.22 |
| Lateral Contact Impulse [%BW*s] | 21.92 ± 13.42 | **21.42 ± 10.45** | 22.85 ± 12.36 | 24.47 ± 13.96 | 33.29 ± 19.04 | 25.96 ± 13.72 | 29.79 ± 14.73 |
| Total Contact Impulse [%BW*s] | 136.51 ± 22.39 | **135.61 ± 18.80** | 139.56 ± 18.90 | 142.51 ± 26.34 | 151.50 ± 40.42 | 147.02 ± 35.69 | 146.25 ± 34.77 |

**Bold** indicates value with greatest reduction.

**Supplementary Table 3.** Comparison of MRI-based and Linearly scaled (LS) models: Individual patient total and lateral compartmental contact peak forces and impulses (mean ± standard deviation from n=3 trials) of each gait alteration.

|  | **Shod** | | **Insoles 0°** | | **Insoles 5°** | | **Insoles 10°** | | **Toe In** | | **Toe Out** | | **Width** | |  |
| --- | --- | --- | --- | --- | --- | --- | --- | --- | --- | --- | --- | --- | --- | --- | --- |
|  | **LS** | **MRI** | **LS** | **MRI** | **LS** | **MRI** | **LS** | **MRI** | **LS** | **MRI** | **LS** | **MRI** | **LS** | **MRI** |  |
| **Peak Lateral Contact Force [%BW]** | | | | | | | | | | | | | | | |
| Patient 1 | 86.41 ± 5.01 | 93.58 ± 3.19 | 90.39 ± 2.73 | 85.17 ± 4.66 | 93.71 ± 5.22 | 95.85 ± 17.23 | 87.89 ± 3.81 | 91.04 ± 8.22 | 99.23 ± 5.90 | 112.09 ± 5.77 | 92.43 ± 3.61 | 78.51 ± 8.17 | 100.04 ± 7.57 | 76.67 ± 10.14 |  |
| Patient 2 | 54.88 ± 5.91 | 54.25 ± 7.82 | 53.11 ± 3.55 | 59.69 ± 1.18 | 51.51 ± 7.60 | 68.47 ± 1.16 | 57.29 ± 3.55 | 65.86 ± 2.89 | 71.25 ± 1.74 | 68.81 ± 0.84 | 65.50 ± 6.20 | 67.43 ± 7.15 | 72.08 ± 9.49 | 64.70 ± 2.09 |  |
| Patient 3 | 64.95 ± 5.25 | 118.91 ± 16.67 | 68.80 ± 3.15 | 110.49 ± 4.32 | 68.93 ± 8.54 | 111.97 ± 24.36 | 72.54 ± 1.82 | 119.46 ± 25.49 | 65.76 ± 2.95 | 134.00 ± 44.79 | 76.81 ± 9.62 | 104.99 ± 2.55 | 73.63 ± 2.99 | 116.04 ± 7.23 |  |
| Patient 4 | 84.97 ± 3.53 | 64.01 ± 4.12 | 85.88 ± 6.86 | 51.98 ± 7.36 | 78.42 ± 3.60 | 53.22 ± 6.93 | 80.78 ± 5.69 | 54.88 ± 7.12 | 90.25 ± 3.67 | 84.27 ± 1.95 | 88.88 ± 3.33 | 101.94 ± 18.42 | 95.67 ± 10.95 | 98.21 ± 6.62 |  |
| Patient 5 | 78.16 ± 5.77 | 81.71 ± 6.94 | 81.81 ± 14.57 | 85.83 ± 15.82 | 103.03 ± 7.19 | 113.63 ± 10.11 | 98.41 ± 5.30 | 108.43 ± 9.50 | 92.43 ± 10.48 | 100.26 ± 9.67 | 71.20 ± 1.02 | 80.86 ± 3.59 | 84.48 ± 6.27 | 80.50 ± 7.30 |  |
| **Peak Total Contact Force [%BW]** | | | | | | | | | | | | | | | |
| Patient 1 | 356.76 ± 13.28 | 341.28 ± 17.67 | 357.50 ± 25.45 | 355.52 ± 27.03 | 362.64 ± 8.40 | 358.29 ± 5.99 | 363.03 ± 4.29 | 351.08 ± 8.04 | 346.64 ± 19.33 | 319.19 ± 18.18 | 328.75 ± 38.26 | 323.53 ± 36.60 | 355.61 ± 23.69 | 341.07 ± 16.93 |  |
| Patient 2 | 258.04 ± 5.15 | 268.05 ± 13.60 | 244.40 ± 3.70 | 266.35 ± 3.95 | 252.85 ± 4.97 | 280.16 ± 0.56 | 252.62 ± 7.94 | 260.97 ± 2.91 | 269.69 ± 1.65 | 271.96 ± 5.79 | 274.22 ± 22.64 | 277.78 ± 20.41 | 258.10 ± 10.45 | 252.18 ± 14.86 |  |
| Patient 3 | 293.82 ± 2.70 | 315.78 ± 29.99 | 300.47 ± 0.42 | 314.79 ± 11.14 | 307.56 ± 19.91 | 316.32 ± 39.19 | 328.89 ± 4.09 | 337.01 ± 36.28 | 298.04 ± 8.89 | 326.56 ± 70.27 | 315.09 ± 22.64 | 292.95 ± 10.77 | 287.06 ± 3.53 | 289.00 ± 13.22 |  |
| Patient 4 | 331.83 ± 17.68 | 298.44 ± 3.14 | 321.67 ± 31.32 | 278.51 ± 3.00 | 296.99 ± 9.16 | 273.00 ± 12.15 | 310.12 ± 11.47 | 281.01 ± 9.03 | 304.27 ± 26.21 | 315.55 ± 15.28 | 297.09 ± 8.62 | 365.20 ± 39.69 | 308.70 ± 36.68 | 358.35 ± 26.33 |  |
| Patient 5 | 253.00 ± 19.06 | 231.32 ± 15.07 | 247.06 ± 3.03 | 231.67 ± 5.87 | 250.10 ± 8.24 | 265.84 ± 22.65 | 252.01 ± 15.76 | 238.33 ± 28.86 | 241.73 ± 7.09 | 223.56 ± 4.07 | 281.99 ± 14.40 | 270.64 ± 18.56 | 248.14 ± 8.20 | 246.31 ± 7.76 |  |

| **Lateral Contact Force Impulse [%BW*s]** | | | | | | | | | | | | | | | |
| --- | --- | --- | --- | --- | --- | --- | --- | --- | --- | --- | --- | --- | --- | --- | --- |
| Patient 1 | 31.09 ± 2.94 | 17.91 ± 2.21 | 29.45 ± 0.85 | 14.95 ± 1.17 | 30.97 ± 1.69 | 17.52 ± 1.47 | 30.33 ± 1.47 | 16.53 ± 1.34 | 35.11 ± 0.39 | 20.36 ± 0.88 | 29.60 ± 1.38 | 13.14 ± 1.83 | 31.57 ± 1.17 | 16.91 ± 1.56 |  |
| Patient 2 | 14.77 ± 2.31 | 15.06 ± 2.00 | 15.75 ± 2.22 | 19.66 ± 1.87 | 17.17 ± 2.36 | 20.39 ± 2.48 | 17.23 ± 1.14 | 19.03 ± 1.20 | 18.34 ± 2.70 | 17.06 ± 2.75 | 13.05 ± 1.70 | 13.07 ± 3.36 | 16.84 ± 2.12 | 14.13 ± 2.09 |  |
| Patient 3 | 12.73 ± 1.61 | 46.45 ± 7.25 | 8.53 ± 3.05 | 40.84 ± 2.79 | 12.47 ± 3.86 | 45.37 ± 6.58 | 13.22 ± 3.01 | 49.66 ± 11.40 | 18.81 ± 1.34 | 61.21 ± 8.07 | 14.43 ± 1.87 | 40.01 ± 1.29 | 13.29 ± 3.47 | 48.10 ± 9.63 |  |
| Patient 4 | 40.39 ± 10.94 | 16.79 ± 7.15 | 37.23 ± 8.14 | 14.86 ± 1.19 | 33.76 ± 2.21 | 18.01 ± 4.13 | 39.91 ± 6.30 | 20.27 ± 3.70 | 50.10 ± 11.89 | 46.67 ± 12.32 | 48.24 ± 3.25 | 42.28 ± 3.70 | 53.51 ± 6.56 | 43.30 ± 2.70 |  |
| Patient 5 | 25.04 ± 3.87 | 13.41 ± 1.74 | 30.08 ± 3.39 | 16.78 ± 4.53 | 24.36 ± 0.52 | 12.97 ± 2.53 | 27.70 ± 1.21 | 16.86 ± 3.77 | 34.46 ± 2.00 | 21.13 ± 2.56 | 27.96 ± 4.79 | 21.31 ± 8.00 | 31.17 ± 1.08 | 26.50 ± 1.12 |  |
| **Total Contact Force Impulse [%BW*s]** | | | | | | | | | | | | | | | |
| Patient 1 | 117.00 ± 6.33 | 119.02 ± 5.27 | 112.77 ± 2.01 | 112.82 ± 1.01 | 115.36 ± 2.50 | 117.43 ± 2.84 | 113.25 ± 3.33 | 115.05 ± 4.34 | 119.72 ± 1.23 | 114.33 ± 3.23 | 111.94 ± 5.09 | 108.96 ± 6.32 | 115.26 ± 0.46 | 116.02 ± 2.09 |  |
| Patient 2 | 131.04 ± 1.95 | 129.63 ± 1.38 | 133.33 ± 2.72 | 134.43 ± 1.88 | 132.54 ± 3.41 | 133.18 ± 4.33 | 135.92 ± 3.12 | 133.27 ± 0.70 | 137.65 ± 1.96 | 134.03 ± 3.05 | 132.29 ± 4.96 | 132.54 ± 3.72 | 128.27 ± 3.19 | 123.15 ± 1.20 |  |
| Patient 3 | 141.86 ± 1.62 | 159.09 ± 9.84 | 140.35 ± 5.03 | 158.96 ± 5.91 | 144.59 ± 6.37 | 162.68 ± 7.16 | 159.90 ± 3.17 | 177.99 ± 15.30 | 157.91 ± 9.06 | 180.46 ± 11.76 | 155.56 ± 6.38 | 158.84 ± 3.03 | 146.93 ± 12.10 | 169.64 ± 15.66 |  |
| Patient 4 | 179.61 ± 17.33 | 162.89 ± 10.46 | 166.97 ± 22.65 | 151.96 ± 7.77 | 156.53 ± 0.81 | 158.44 ± 3.36 | 171.75 ± 13.93 | 164.87 ± 5.78 | 175.84 ± 13.33 | 211.80 ± 14.81 | 187.07 ± 7.25 | 207.22 ± 4.67 | 187.63 ± 11.37 | 199.67 ± 9.65 |  |
| Patient 5 | 129.83 ± 8.32 | 111.94 ± 5.33 | 138.78 ± 2.39 | 119.89 ± 2.09 | 140.13 ± 2.66 | 126.09 ± 2.32 | 137.26 ± 3.97 | 121.38 ± 1.76 | 138.87 ± 4.33 | 116.88 ± 4.53 | 137.60 ± 8.73 | 127.54 ± 11.48 | 130.80 ± 4.87 | 122.77 ± 4.46 |  |

*Blue* boarder indicates greatest reduction in LS trials and *yellow* boarder indicates greatest reduction in MRI trials.

**Supplementary Table 4.** Comparison of MRI-based and Linearly scaled (LS) models: Individual patient Knee flexion angle (KFA) range of motion (ROM), peak Knee flexion moments (KFM), and KFM impulses (mean ± standard deviation from n=3 trials) of each gait alteration.

|  | **Shod** | | **Insoles 0°** | | **Insoles 5°** | | **Insoles 10°** | | **Toe In** | | **Toe Out** | | **Width** | |
| --- | --- | --- | --- | --- | --- | --- | --- | --- | --- | --- | --- | --- | --- | --- |
|  | **LS** | **MRI** | **LS** | **MRI** | **LS** | **MRI** | **LS** | **MRI** | **LS** | **MRI** | **LS** | **MRI** | **LS** | **MRI** |
| **Knee Flexion Angle ROM [°]** | | | | | | | | | | | | | | |
| Patient 1 | 48.43 ± 0.75 | 39.67 ± 1.15 | 47.10 ± 0.93 | 38.29 ± 1.28 | 48.43 ± 1.58 | 39.19 ± 1.03 | 46.35 ± 1.15 | 37.10 ± 1.06 | 47.15 ± 1.48 | 38.02 ± 1.35 | 48.93 ± 1.25 | 40.62 ± 1.02 | 43.05 ± 2.32 | 35.28 ± 1.66 |
| Patient 2 | 40.09 ± 1.44 | 40.86 ± 1.26 | 39.67 ± 0.87 | 41.51 ± 0.99 | 40.83 ± 2.22 | 42.67 ± 2.15 | 42.19 ± 3.19 | 43.74 ± 3.12 | 38.35 ± 1.55 | 39.24 ± 1.98 | 39.02 ± 1.88 | 39.96 ± 1.65 | 39.91 ± 0.30 | 40.46 ± 0.46 |
| Patient 3 | 37.29 ± 2.10 | 40.86 ± 2.60 | 38.74 ± 0.77 | 42.49 ± 0.39 | 36.97 ± 0.18 | 40.50 ± 0.40 | 38.22 ± 0.75 | 41.85 ± 0.55 | 33.64 ± 2.04 | 38.97 ± 1.83 | 38.64 ± 0.59 | 42.43 ± 0.87 | 34.31 ± 0.92 | 37.18 ± 1.05 |
| Patient 4 | 47.45 ± 2.54 | 46.38 ± 2.28 | 47.77 ± 0.49 | 46.22 ± 0.74 | 46.80 ± 0.33 | 44.68 ± 0.24 | 44.83 ± 0.70 | 43.07 ± 0.53 | 41.59 ± 3.76 | 42.21 ± 2.75 | 45.56 ± 0.54 | 43.39 ± 0.26 | 43.23 ± 1.06 | 41.42 ± 0.88 |
| Patient 5 | 31.26 ± 3.14 | 29.10 ± 3.66 | 31.03 ± 0.24 | 28.40 ± 0.15 | 32.18 ± 0.80 | 30.04 ± 0.50 | 29.90 ± 0.17 | 29.09 ± 0.66 | 30.60 ± 1.91 | 28.43 ± 1.16 | 30.45 ± 0.64 | 30.55 ± 0.77 | 27.63 ± 0.96 | 25.22 ± 1.60 |
| **Peak KFM [%BW*BH]** | | | | | | | | | | | | | | |
| Patient 1 | 2.27 ± 0.06 | 1.61 ± 0.09 | 2.05 ± 0.02 | 1.39 ± 0.03 | 2.28 ± 0.01 | 1.69 ± 0.08 | 2.25 ± 0.04 | 1.58 ± 0.04 | 2.39 ± 0.11 | 1.69 ± 0.10 | 2.08 ± 0.02 | 1.31 ± 0.14 | 2.15 ± 0.05 | 1.44 ± 0.12 |
| Patient 2 | 2.44 ± 0.06 | 2.53 ± 0.07 | 2.18 ± 0.16 | 2.61 ± 0.12 | 2.44 ± 0.07 | 2.87 ± 0.03 | 2.09 ± 0.08 | 2.41 ± 0.13 | 2.72 ± 0.07 | 2.88 ± 0.12 | 2.14 ± 0.10 | 2.18 ± 0.21 | 2.30 ± 0.42 | 2.28 ± 0.42 |
| Patient 3 | 2.34 ± 0.10 | 3.01 ± 0.30 | 2.18 ± 0.08 | 2.88 ± 0.11 | 2.25 ± 0.29 | 2.79 ± 0.41 | 2.17 ± 0.32 | 2.83 ± 0.63 | 2.49 ± 0.23 | 3.19 ± 0.74 | 2.09 ± 0.20 | 2.55 ± 0.30 | 2.12 ± 0.12 | 2.43 ± 0.16 |
| Patient 4 | 2.92 ± 0.54 | 1.94 ± 0.31 | 3.05 ± 0.17 | 1.78 ± 0.18 | 2.99 ± 0.17 | 1.72 ± 0.10 | 3.13 ± 0.35 | 1.85 ± 0.22 | 3.01 ± 0.39 | 2.37 ± 0.34 | 2.96 ± 0.32 | 2.19 ± 0.45 | 3.22 ± 0.51 | 2.25 ± 0.31 |
| Patient 5 | 1.91 ± 0.05 | 1.46 ± 0.21 | 1.84 ± 0.07 | 1.40 ± 0.14 | 2.14 ± 0.24 | 1.89 ± 0.23 | 2.08 ± 0.20 | 1.82 ± 0.31 | 1.82 ± 0.06 | 1.57 ± 0.08 | 2.01 ± 0.11 | 1.59 ± 0.17 | 2.23 ± 0.02 | 1.85 ± 0.17 |
| **KFM Impulse [%BW*BH*s]** | | | | | | | | | | | | | | |
| Patient 1 | 0.57 ± 0.04 | 0.40 ± 0.03 | 0.49 ± 0.03 | 0.32 ± 0.02 | 0.56 ± 0.02 | 0.39 ± 0.01 | 0.55 ± 0.04 | 0.37 ± 0.04 | 0.61 ± 0.04 | 0.43 ± 0.03 | 0.51 ± 0.02 | 0.30 ± 0.02 | 0.54 ± 0.03 | 0.34 ± 0.04 |
| Patient 2 | 0.90 ± 0.03 | 0.70 ± 0.05 | 0.84 ± 0.06 | 0.71 ± 0.08 | 0.85 ± 0.06 | 0.73 ± 0.06 | 0.76 ± 0.04 | 0.60 ± 0.05 | 0.99 ± 0.04 | 0.82 ± 0.05 | 0.81 ± 0.03 | 0.57 ± 0.06 | 0.83 ± 0.07 | 0.59 ± 0.09 |
| Patient 3 | 0.81 ± 0.07 | 0.97 ± 0.16 | 0.75 ± 0.09 | 0.91 ± 0.10 | 0.74 ± 0.05 | 0.89 ± 0.07 | 0.84 ± 0.11 | 1.01 ± 0.20 | 0.99 ± 0.14 | 1.22 ± 0.27 | 0.88 ± 0.04 | 0.99 ± 0.08 | 0.71 ± 0.07 | 0.86 ± 0.09 |
| Patient 4 | 1.44 ± 0.25 | 0.73 ± 0.22 | 1.39 ± 0.20 | 0.69 ± 0.06 | 1.26 ± 0.04 | 0.65 ± 0.07 | 1.48 ± 0.25 | 0.80 ± 0.17 | 1.43 ± 0.21 | 0.80 ± 0.23 | 1.72 ± 0.16 | 1.03 ± 0.16 | 1.73 ± 0.22 | 1.07 ± 0.09 |
| Patient 5 | 0.80 ± 0.04 | 0.48 ± 0.11 | 0.80 ± 0.02 | 0.43 ± 0.03 | 0.79 ± 0.03 | 0.46 ± 0.07 | 0.84 ± 0.05 | 0.50 ± 0.11 | 0.84 ± 0.06 | 0.49 ± 0.05 | 0.74 ± 0.04 | 0.40 ± 0.05 | 0.80 ± 0.06 | 0.53 ± 0.04 |

*Blue* boarder indicates greatest reduction in LS trials and *yellow* boarder indicates greatest reduction in MRI trials.
