# Supplementary figures and images for: Gait alteration strategies for knee osteoarthritis: a comparison of joint loading via generic and patient-specific musculoskeletal model scaling techniques
Source: Int Biomech. 2019 Jul 21;6(1):54–65. doi: 10.1080/23335432.2019.1629839 (PMC7857308; doi:10.1080/23335432.2019.1629839)

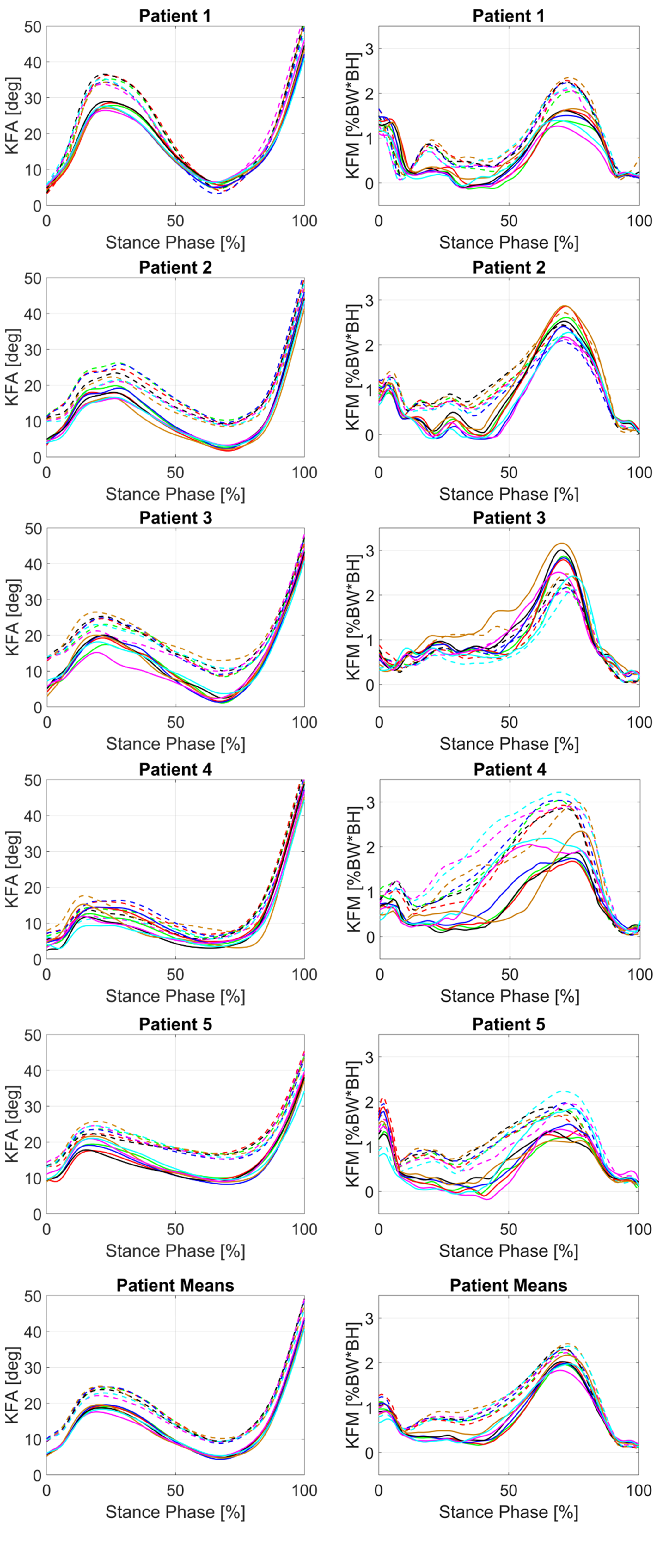

Supplement: Supplemental Material [file TBBE_A_1629839_SM1759.zip › SupplementaryFigure1 - Compressed 300 x 300.png]
